# A novel method to measure regional muscle blood flow continuously using NIRS kinetics information

**DOI:** 10.1186/1476-5918-5-5

**Published:** 2006-05-16

**Authors:** Shoko Nioka, Ryotaro Kime, Ulas Sunar, Joohee Im, Meltem Izzetoglu, Jun Zhang, Burak Alacam, Britton Chance

**Affiliations:** 1 Department of Biochemistry and Biophysics, Medical School of University of Pennsylvania, Philadelphia, PA 19104, USA

## Abstract

**Background:**

This article introduces a novel method to continuously monitor regional muscle blood flow by using Near Infrared Spectroscopy (NIRS). We demonstrate the feasibility of the new method in two ways: (1) by applying this new method of determining blood flow to experimental NIRS data during exercise and ischemia; and, (2) by simulating muscle oxygenation and blood flow values using these newly developed equations during recovery from exercise and ischemia.

**Methods:**

Deoxy (Hb) and oxyhemoglobin (HbO_2_), located in the blood ofthe skeletal muscle, carry two internal relationships between blood flow and oxygen consumption. One is a mass transfer principle and the other describes a relationship between oxygen consumption and Hb kinetics in a two-compartment model. To monitor blood flow continuously, we transfer these two relationships into two equations and calculate the blood flow with the differential information of HbO_2 _and Hb. In addition, these equations are used to simulate the relationship between blood flow and reoxygenation kinetics after cuff ischemia and a light exercise. Nine healthy subjects volunteered for the cuff ischemia, light arm exercise and arm exercise with cuff ischemia for the experimental study.

**Results:**

Analysis of experimental data of both cuff ischemia and light exercise using the new equations show greater blood flow (four to six times more than resting values) during recovery, agreeing with previous findings. Further, the simulation and experimental studies of cuff ischemia and light exercise agree with each other.

**Conclusion:**

We demonstrate the accuracy of this new method by showing that the blood flow obtained from the method agrees with previous data as well as with simulated data. We conclude that this novel continuous blood flow monitoring method can provide blood flow information non-invasively with NIRS.

## Background

Near Infrared Spectroscopy (NIRS) has been used to measure regional skeletal muscle deoxygenation [1-4] by presenting a balance between oxygen delivery and demand in muscle tissue. NIRS is beneficial since it is non-invasive and does not restrict ongoing activities at sports events such as skiing [5] and skating [6]. Further, it is used to measure local muscular tissue circulation, namely blood flow [7] and oxygen consumption (O_2_) [8]. The validity of these measurements and their relationship to global metabolism has been well established [9,10]. Thus, NIRS technology contributes to the understanding of regional muscular metabolism and O_2 _transport systems.

NIRS is a unique method since hemoglobin in smaller vessels can be detected with greater sensitivity than it can be in larger vessels when the probe is located on the surface of a muscle. Most of the hemoglobin information in NIRS comes from vessels less than 2 mm [11]. This sensitivity feature of NIRS favors the measurement of the microvasculature of regional muscle. On the other hand, it is difficult to predict the hemoglobin distribution ratio between artery, capillary and vein. Nevertheless, NIRS measurements are well validated and are generally consistent with other methods [7-10,12]. Oxygen consumption and blood flow of regional muscles from NIRS (venous and arterial occlusion methods) have been compared to more global methods, large arterial flow using Doppler ultrasound method and global tissue flow using plethysmography, and differences have been found. For example, NIR-determined regional muscle blood flow change more with exercise than with global blood flow, while other global methods show a greater initial value and change less than with the NIRS method. This can be understood since measurements from global methods are averaged from other tissues, such as bones and skin or other neighboring muscles. It is clear that the NIRS method measures more regional blood flow from the microvasculature of a muscle than those methods using large arteries and global tissues. In addition, since NIRS targets red cell flow in the microvasculature and those are directly related to oxygen availability, NIRS based blood flow is a more important and closer parameter for O_2 _transport than large arterial blood flow. Therefore it is beneficial to develop an accurate method of regional blood flow measurement using NIRS. However current NIRS blood flow measurement by a venous occlusion maneuver, results in intermittent measurements and it is not always desirable to do occlusion every 10 seconds. Other technologies used for monitoring blood flow – Doppler ultrasound [13], MRI [14], and PET [15] – can also be troublesome since they require many restrictions on muscle performance and are not always available. Therefore, it is desirable to find a way to measure blood flow during muscle exercise without disturbing muscle performance during sports events.

In this investigation, we introduce a novel method that measures blood flow using information of tissue blood hemoglobin available with NIRS. The blood flow determination is achieved by calculating blood flow from the following two equations using NIR data on deoxy-hemoglobin (Hb) and oxy-hemoglobin (HbO_2_) : 1) a mass transfer equation of [HbO_2_] and 2) an equation that describes the rate of appearance of Hb as exactly analogous to the oxygen consumption rate, O_2_, with a two-compartment model of hemoglobin distribution. Similar equations are derived to describe 1) isometric exercises at the onset of oxygenation as an indication of O_2 _using a three-compartment model [16]; and 2) inflow and outflow hemodynamics due to the effects of external compression on the human breast with a two-compartment model [17]. In addition, since these two equations describe the relationships among blood flow, oxygen consumption and muscle tissue oxygenation, we also use them for a simulation tool.

Thus, in this study, we introduce equations to describe blood flow with Hb and HbO_2 _kinetics and O_2 _in a two-compartment model. Then, we apply measured concentrations of Hb and HbO_2 _([Hb] and [HbO_2_]) in arm muscles from experimental NIRS data to calculate blood inflow/outflow, observed in cuff ischemia and light exercise. Further, using the equations, we simulate and describe relationships between blood flow and reoxygenation kinetics during arm cuff ischemia and exercise. Finally, we compare calculated blood flow using measured differential information of [Hb] and [HbO_2_] to simulated cases, as well as to previous findings, and find good agreement among them. Thus, we demonstrate feasibility of the novel method to monitor regional muscle blood flow continuously by NIRS.

## Methods

### Principle of continuous blood flow measurement using [HbO_2_] and [Hb] kinetics

The principle of blood flow measurement is based on differential information of [Hb] and [HbO_2_] obtained by the NIRS. The NIRS data can be obtained by placing the NIRS probe on a muscle of interest and conducting an experiment, such as cuff ischemia and light exercise, that causes changes in the muscle [Hb] and [HbO_2_]. The blood flow calculations are based on a two-compartment model, where all the blood in the tissue belongs to either an arterial or venous compartment including capillaries. The first equation describes mass conservation in the two compartments of the muscle.

*d*[*HbO*_2_]/*dt *= *Q*_a _* *S*_*a *_*O*_2 _- *Q*_v _* *S*_*v *_*O*_2 _- *O*_2 _    (1)

[HbO_2_] is a concentration of HbO_2 _(μM/kg tissue), Q_a _and Q_v _are arterial blood flow and venous blood flow respectively, expressed as hemoglobin mass as μMTHb/sec/kg tissue or μMTHb/sec/kg. O_2 _is the metabolic rate of the tissue, expressed as oxyhemoglobin carrying capacity μMHbO_2_/sec/kg tissue. Note that these two parameters, Q and O_2_, share the same units, namely hemoglobin concentration (total or oxyhemoglobin) per second in a kg tissue. This is similar to comparing O_2 _availability of Q and O_2 _utilization of O_2_, using the same unit of either ml O_2_/min/100 g or μM O_2_/min/100 g. (The translation of 1μM hemoglobin O_2 _carrying capacity is equivalent to 4 μM of O_2_, and 1 μM Hb O_2_/sec/kg of either Q and O_2 _translates to 24 μM O_2_/min/100 g, or 0.54 ml O_2_/min/100 g (O_2_) and 3 ml blood/min/100 g (Q).)

S_v_O_2 _is hemoglobin saturation with the oxygen in the venous blood:



Likewise S_a_O_2 _is hemoglobin saturation with the oxygen in the arterial blood, which is assumed to be 100%.

Equation 3 explains that oxygen is extracted from oxyhemoglobin, which is converted to deoxyhemoglobin with the same rate as O_2 _and some is lost through venous outflow.

*d*[*Hb*]/*dt *= *O*_2 _- *Q*_*v *_(1 - *S*_*v *_*O*_2_)     (3)

With the equations (1), (2), and (3), we come to the solutions of Q_a _and Q_v _as follows;





[HbO_2_]_a _represents HbO_2 _in the arterial blood, and [HbO_2_] represents total oxyhemoglobin ([HbO_2_] = [HbO_2_]_a _+ [HbO_2_]_v_). Equations (4) and (5) demonstrate that we can calculate the blood flow of the tissue, inflow and outflow, separately with [HbO_2_], [Hb], and [HbO_2_]_a_, as well as the changes of [HbO_2_] and [Hb], and O_2_. The necessary normal resting values of muscle tissue [Hb] and [HbO_2_] in the equations can be measured by a frequency domain (FD) device (IQ system [18,19]) in arm finger flexor muscles. O_2 _information can be also measured by the slope of HbO_2 _disappearance during the cuff treatment [8].

The initial state of resting muscle is under steady state, therefore inflow is same as outflow (Q_a _= Q_v_). The inflow and outflow of the arm muscle, Q_a _and Q_v _correlate directly with THb, ([HbO_2_] + [Hb]) and changes in the following equation;

dTHb/dt = Q_a _- Q_v _    (6)

Equation 6 states that the changes in THb are attributed to hyperemia, the imbalance between Q_a _and Q_v _that is typically seen in the recovery phase of either ischemia or exercise. Both situations occur due to the hypoxic status of the muscle tissue prior to the hyperemia [20]. Oxygenation is defined by

Oxygenation = [HbO_2_] - [Hb]     (7)

**Figure 1:**
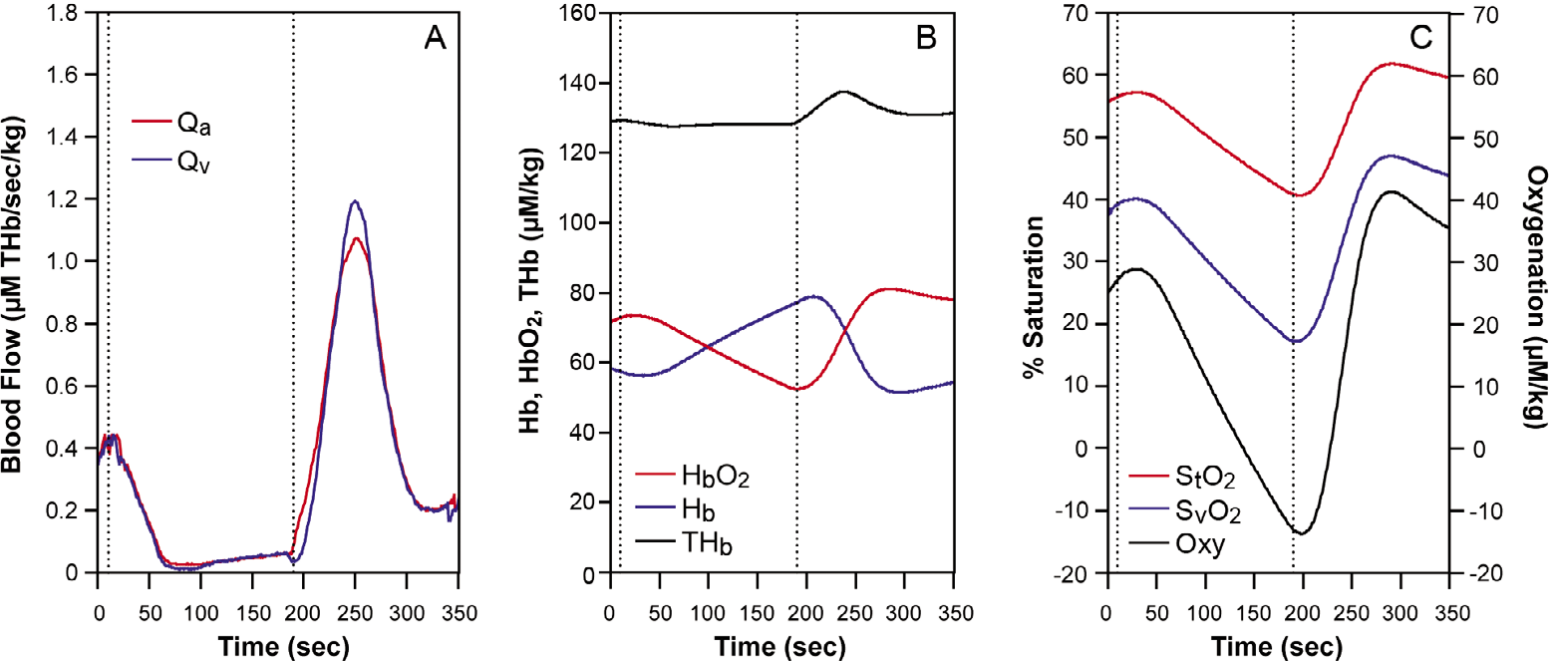
A healthy subject arm ischemia data. The cuff was imposed between two lines. (A) blood flow, Q_a_: red, Q_v_: blue(THb μM/sec/kg). (B) NIRS data, HbO_2_: red, Hb: blue, THb: black (μM/kg). (C) tissue saturation, %S_t_O_2_: red, venous saturation, %S_v_O_2_: blue, Oxygenation: black. (μM/kg).

**Figure 2:**
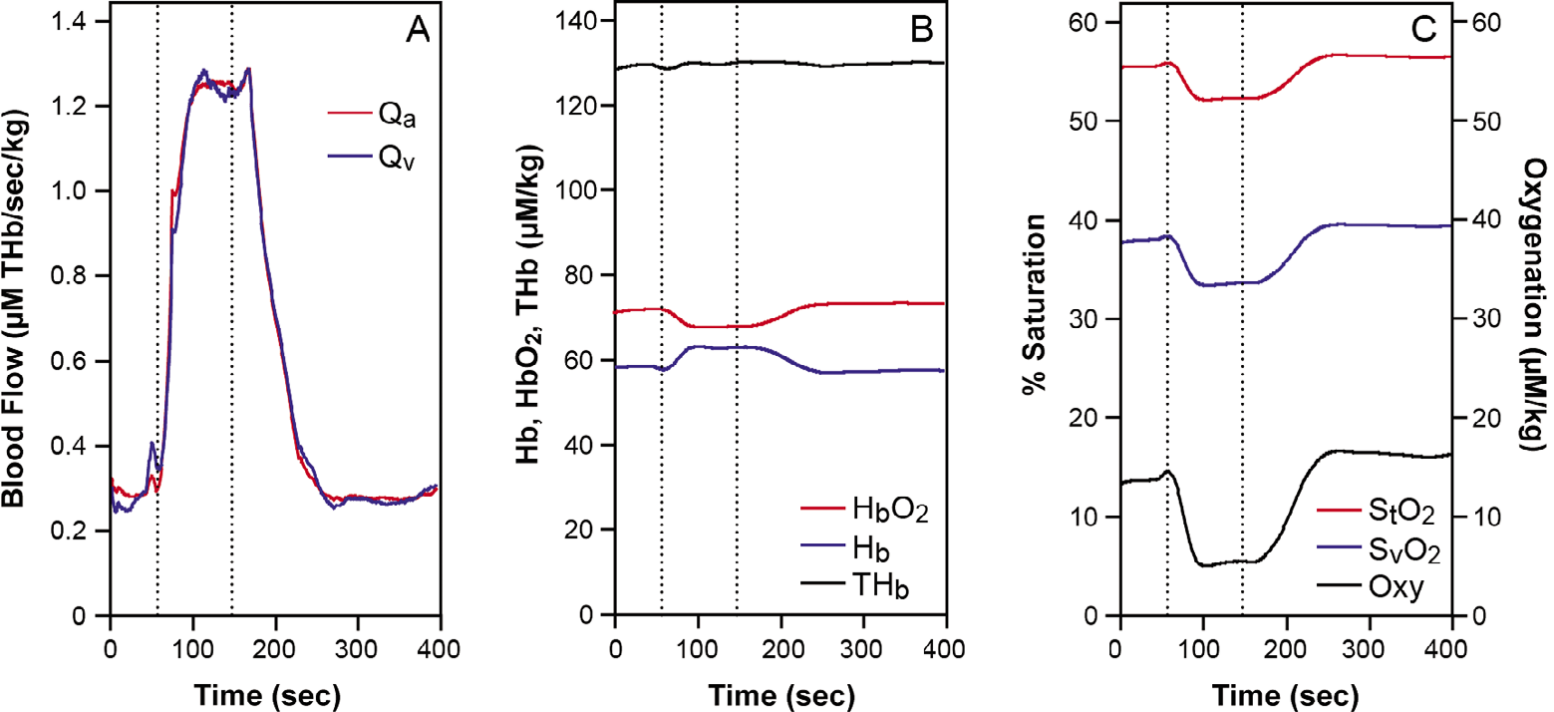
A healthy human arm data during handgrip exercise (between two lines) at 25% MVC for 90 seconds, (A), Q_a_: red, Q_v_: blue (THb μM/sec/kg). (B), HbO_2_: red, Hb: blue, THb: black (μM/kg). (C), %S_t_O_2_: red, %S_v_O_2_: blue, Oxygenation: black (μM/kg).

**Figure 3:**
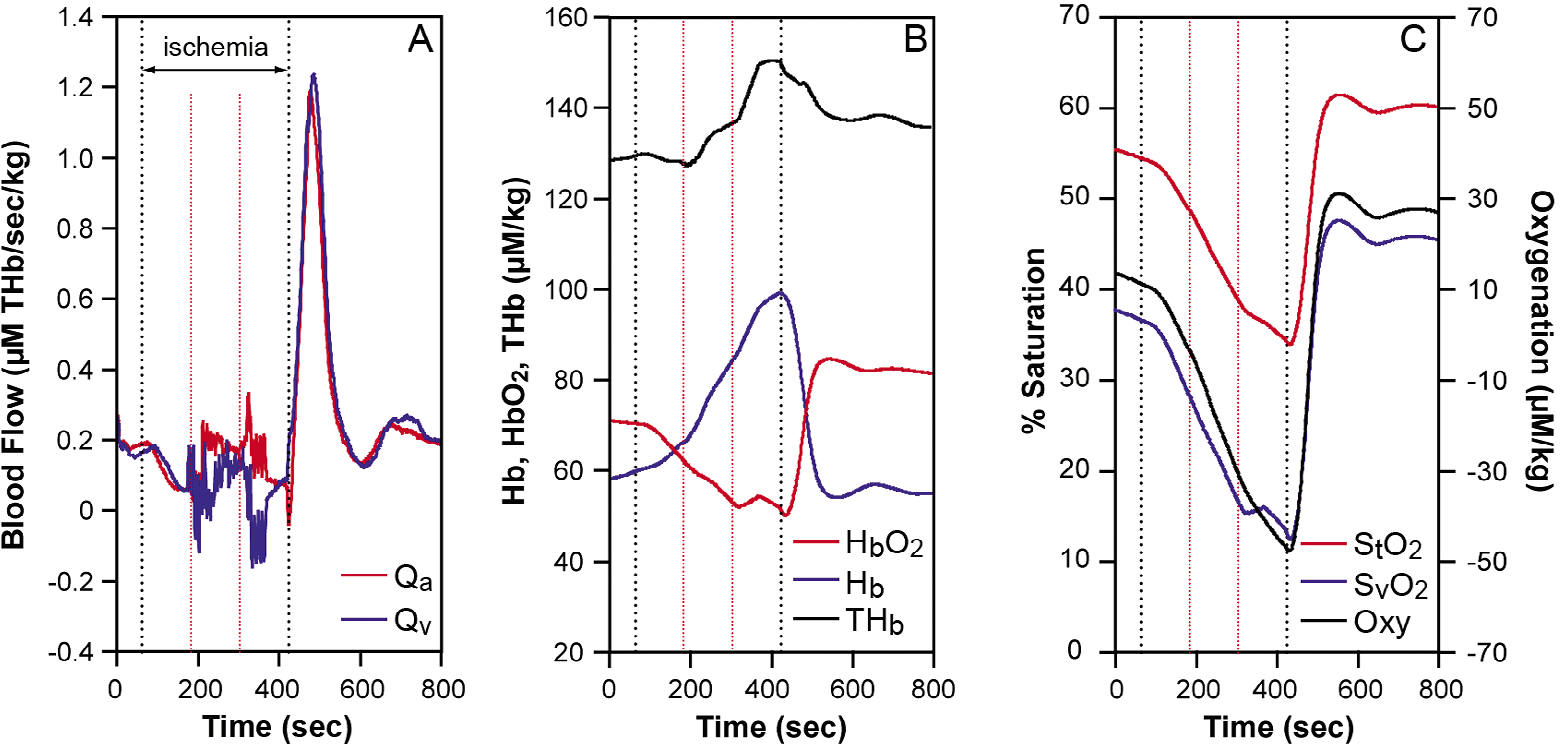
A healthy subject ischemic exercise data (between red lines) at 75% MVC. In the 6 min ischemia (between black lines), at 100 seconds to 220 sec (resting), exercise (220 to 340 sec) and resting (340 to 460 sec) with the cuff on. (A), Q_a_: red, Q_v_: blue, (THb μM/sec/kg). (B), HbO_2_: red, Hb: blue, BV: black (μM/kg). (**C**), %S_t_O_2_: red, %S_v_O_2_: blue, Oxygenation: black (μM/kg).

**Figure 4:**
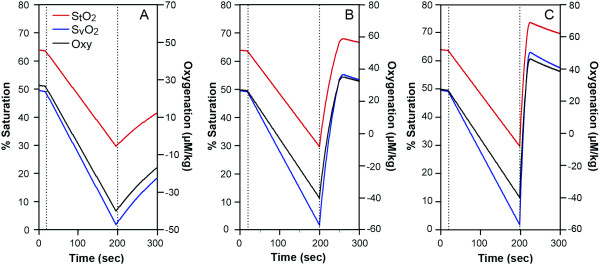
Simulation of arm ischemia model (arm cuff between lines) with various recovery blood flows. Reoxygenation kinetics are shown as %S_t_O_2_: red, %S_v_O_2_: blue and oxygenation: black (μM/kg). Various blood flows during the recovery; (A), resting flow, (B), 4 times of resting, (C), ten times as high as resting, were inputted into the model that made the various reoxygenation kinetics.

**Figure 5:**
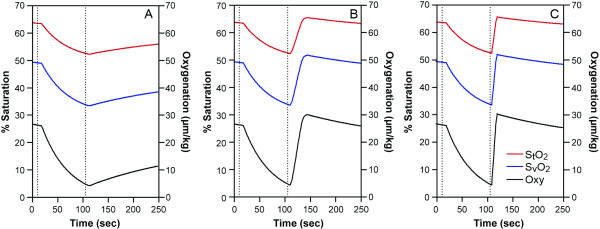
Simulation of 90 seconds handgrip exercise (between lines) of 25% MVC with 5 times O_2 _and 4 times blood flow of the resting, followed by various blood flow during the recovery phase. The variable recovery blood flows are: (A) the same as the resting value, (B), 4 times of the resting value, and (C), 10 times of the resting value. %S_t_O_2_: red, %S_v_O_2_: blue, oxygenation: black (μM/kg). The reoxygenation rate during the recovery increases with greater blood flow (from A to C).

Oxygenation (μM/kg) is used as an indicator of relative oxygen saturation of the muscle tissue when the absolute values of saturations are not known [1], and can be negative value. Oxygenation values show how the simple expression of Oxygenation ([HbO_2_] - [Hb]) can represent relative values of saturation by comparison to StO_2 _and SvO_2 _in figures describing experimental data (Figures 1, 2, 3) as well as simulation (Figures 4, 5).

StO_2 _= [HbO_2_]/THb     (8)

### Subjects and materials

The protocol was approved by the IRB of the University of Pennsylvania. Nine healthy volunteers aged from 21 to 30 years agreed to perform an arm cuff ischemia for three minutes with NIRS (IQ system, NIM incorporated, Philadelphia), which served as initial resting muscle parameters, as well as continuous time spectra of [HbO_2_] and [Hb]. Six of them also performed the cuff ischemia followed by a 90-second hand grip exercise with a continuous wave spectrometer (CWS) NIR device. Cuff ischemia was imposed for three minutes in the forearm at the pressure of 220 mmHg and a five minute recovery time period was observed. The light arm grip test was 90 seconds at the 25% MVC with a frequency of 0.5 Hz. The work intensities were determined by a dynamometer. We also did arm exercise at 75% MVC (0.5 Hz) with cuff in 3 subjects.

Frequency Domain NIRS was used to calculate absolute resting arm tissue concentrations of HbO_2_, Hb, S_t_O_2 _and VO_2 _by arterial occlusion, prior to the ischemia, exercise and ischemic exercise protocols, according to absorption and scattering coefficients [21]. The CWS was used during the protocols, and used a 3-wavelengths with light emitting diodes at 735, 805 and 850 nms, which in turn measured Hb and HbO_2 _concentration changes of the hand grip muscle tissues – Δ[Hb] and Δ[HbO_2_] [22].

### Data analysis

The blood flow calculation from each individual needs resting values of [HbO_2_], [Hb] in each subject. To convert the Δ[Hb] and Δ[HbO_2_] obtained with CWS to the absolute concentration, [Hb] and [HbO_2_] in the cuff ischemia and exercise data, we add the values of the resting arm muscles measured by the IQ system to the CWS data. CWS data are smoothed with a 3^rd ^order fitting (Matlab) and expressed as Δ[Hb] and Δ[HbO_2_]. Then the time course of CWS is analyzed for Q_a _and Q_v_. Another necessary parameter for Q_a _and Q_v _is O_2_, and in ischemia protocol, it is provided by the slope of HbO_2_, O_2 _is assumed to be unchanged throughout the measurement. However in the exercise protocol, O_2 _is assumed to have increased 5 times [23]. The arterial and venous [HbO_2_] are also necessary for blood flow calculation and are calculated from the venous blood volume ratio of 28.3%/71.7%. This ratio comes from the venous blood saturation that is assumed to be near 49.1% [24].

For the simulation, we use mean values measured from the 9 healthy subjects with IQ systems, shown in Table 1. The measured mean resting hemoglobin related values from the 9 healthy subjects are [Hb] and [HbO_2_] as 35.8 and 62.3 μM/kg, respectively, blood volume ([Hb] + [HbO_2_]) 98.1 μM/kg, and tissue saturation as 63.5%. The oxygen consumption of 0.185 μM HbO_2_/sec/kg is analogous to 0.1 ml O_2_/min/100 g, and is similar to published data [12]. These are used as the initial resting values for the simulation. All the other necessary values used for the simulations are also listed in Table 1, with the assumptions stated in the comments. We assume hemoglobin concentration of 12.8 g in 100 ml of blood, and that each gram of hemoglobin binds to 1.31 ml of O_2_, 0.3 ml of dissolved O_2 _in the 100 g of blood. The Q_a_, Q_v _values of 0.33 μM THb/sec/kg are analogous to 0.99 ml blood/min/100 g tissue, which is similar to published data [12].

**Table 1:** Parameters for simulation in the resting arm finger flexor muscles

Term	Definition	Values	Unit	comments
THb	total hemoglobin ([HbO2] + [Hb])	98.1 ± 9.3	μM/kg	1
[HbO_2_]	total HbO_2_	62.3 ± 6.0	μM/kg	1
[HbO_2_]_a_	arterial HbO_2_	27.7	μM/kg	2
[HbO_2_]_v_	venous HbO_2_	34.6	μM/kg	2
[Hb]	total deoxyhemoglobin	35.8 ± 4.3	μM/kg	1
O_2_	O_2 _consumption	0.185 ± 0.012	μMHbO_2_/sec/kg	1
Q_a_, Q_v_	arterial and venous blood flow	0.276	μMTHb/sec/kg	3
S_a_O_2_	arterial saturation	100	%	4
S_v_O_2_	venous saturation	49.1	%	5
StO_2_	tissue saturation	63.5 ± 2.1	%	1
a/v ratio	arterial venous blood volume ratio	28.3%/71.7%	%/%	2

Comment 1. These values are from NIR measurements using IQ system (mean ± standard error, n = 9).

Comment 2. These are calculated from S_v_O_2 _and S_t_O_2 _which gives arterial venous blood volume ratio of 28.3%/71.7%.

Comment 3. The blood flow is calculated from Eqs. 4–5.

Comment 4. S_a_O_2 _is assumed 100%.

Comment 5. S_v_O_2 _is assumed 49.1% ([24]).

In the exercise model, we assume the 25%MVC finger flexor exercise produced five times more O_2 _than resting value. At the end, the O_2 _recovered to the resting state within 20 seconds. Then we simulate 3 conditions of recovery blood flow; 1) same as resting, 2) 4 times, and 3) 10 times the resting blood flow. Matlab software is used to run the simulation algorithms and fitting procedures.

## Results

### Continuous measurements of blood flow using kinetic information of NIRS

#### 1. Blood flow during arm ischemia in normal healthy subjects

Typical results from a three minute arm cuff ischemia in healthy subjects are shown in Figure 1. The resting values of [THb], [HbO_2_], [Hb], are 128, 70.3, and 57.5 μM/kg respectively measured by the IQ system and StO_2 _is 55%, O_2 _is 0.2 μM HbO_2_/sec/kg. Average values of muscle hemoglobin in the 6 healthy subjects are listed in Table 1. The healthy arm was cuffed at 220 mmHg, from 10 seconds to 190 seconds, and then, the cuff was released. Blood flow (Q_a_, Q_v_) became near zero during the cuff occlusion and abruptly increased immediately after the release of the cuff. The increase in the blood flow during the recovery phase is from 0.35 to over 1.1 μM THb/sec/kg, about three to four times greater than that of the resting blood flow (Figure 1A). The corresponding reoxygenation can be seen in HbO_2 _(Figure 1B) as well as saturation recovery profiles (Figure 1C). Interestingly, the arterial flow (Q_a_) kinetics is different from that of venous flow (Q_v_). BV increase during the recovery (hyperemia, Figure 1B, black) resulting from the discrepancy of the kinetics between Q_a _and Q_v _(see Equation6). Overall half-time recovery of reoxygenation i**s **approximately 25 seconds.

The high compensatory peak blood flow in the recovery from cuff ischemia for six healthy subjects is on average 4.2 ± 1.9 times of resting values and last for about two minutes and half recovery time of saturation is about 21.2 ± 4.5 seconds, on average. The reoxygenation kinetic phase form is similar to previous findings during arm cuff occlusion [25-27].

#### 2. Continuous blood flow measurement of exercised arm muscle during a 25%MVC handgrip test observed with a NIRS system

We tested the six healthy subjects with the 90 second handgrip test with NIRS. Figure 2 shows typical results of the blood flow of a subject during the handgrip test. The results showed that Q_a_, Q_v _are four to five times greater than the resting value during and at the end of the exercise lasting 15 to 30 more seconds (Figure 2A as an example) in 6 subjects. The half recovery reoxygenation from the exercise is between 15 to 35 seconds (for example see Figure 2B,2C) in 6 subjects. This data agrees with previous studies [23,25,28,29].

#### 3. Continuous blood flow measurement during ischemic exercise

We do not have to assume O_2 _to obtain Q_a _and Q_v _during the ischemic exercise protocol since we are able to calculate O_2 _by taking the initial slope of HbO_2 _during ischemic conditions from the NIRS spectra[8] and inserting O_2 _into the equations provided (Equations 4 and 5). Therefore, the calculation of blood flow (Q_a _and Q_v_) is more quantitative (Figure 3), than in the regular exercise cases, where in Figure 2 for example, we rely on an assumption of O_2_. Figure 3 shows an ischemic exercise of a healthy a subject. During 6 min. ischemia (time between 70 seconds and 430 seconds), after resting, handgrip exercise was imposed (between 180 seconds to 340 seconds). There is some flow during exercise even though the cuff pressure is 220 mmHg. The slight blood flow during the cuff ischemia can be confirmed by increases in the blood volume and HbO_2 _(Figure 3B). The greater blood volume increase is due to venous occlusion with some arterial flow leaking into the tissue. After the cuff ischemia, high blood flow is seen for 100 seconds and the peak blood flow increases as much as six times from the resting blood flow. The high blood flow in the recovery is consistent with studies of the similar protocols [23,25]. The reoxygenation rate **is **around 20 seconds.

### Simulation of the blood flow, and its relationship to reoxygenation kinetics

#### 1. Simulation of blood flow and reoxygenation in the recovery of the arm ischemia

The literature has demonstrated that the blood flow in the recovery from the ischemia is about four to eight times greater than the resting values [25-27]. Our simulation of cuff ischemia includes recovery blood flow values ranging from no compensation (resting value) to over-compensatory flow (ten times of resting value) in the recovery phase from the arm cuff ischemia. We simulate a situation where O_2 _and BV are maintained throughout the experiment for simplicity. Blood flow is completely stopped in the cuff ischemia and returned to parametric values in the recovery stage, from low (resting value, Figure 4A) to middle and high blood flow (four and ten times as high as the resting values, Figure 4B,4C respectively). The duration of the middle and high blood flows in the recovery is 50 seconds and 23 seconds respectively in order to produce muscle reoxygenation to reach a little above the resting values. The results shown in Figure 4 indicate that the parametric blood flow in the recovery from the cuff ischemia alters the reoxygenation rate. The higher flow in the recovery caused the shorter recovery reoxygenation rates.

When the recovery blood flow from the cuff ischemia is the same as that of resting, the reoxygenation rate is extremely slow (half recovery time of approximately three minutes, Figure 4A). On the other hand, the very high flow during the recovery phase (ten times as high as resting, Figure 4C) results in a faster S_t_O_2_, S_v_O_2 _reoxygenation rate and a half-recovery time of six seconds. In the middle of the two extreme cases, the blood flow is four times larger than the resting value (Figure 4B) and the recovery oxygenation rate is approximately 20 seconds for half-recovery time. This simulates the situation of a healthy sedentary arm, where the blood flow function is adequate.

#### 2. Simulation of the blood flow and the reoxygenation rate in the arm handgrip exercise model using NIRS

We simulate the muscle deoxygenation and recovery of Hb and HbO_2 _profiles during 90 seconds handgrip exercise at 25% MVC. Van Beekvelt et al showed that the handgrip exercise test with 25% MVC required approximately five times of resting O_2 _and four times of resting blood flow [27]. They also show some post exercise O_2 _and we assume similar trends during the first part of recovery (O_2 _decay takes 20 seconds in the recovery). We input these values of O_2 _and blood flow during the exercise and recovery phase in a simulation algorithm and treat the blood flow during the recovery as a variable parameter. The recovery blood flow range from the same as resting value (Figure 5A) to 4 four times (Figure 5B) and ten times (Figure 5C) of resting value, and we observe the recovery oxygenation kinetics of the arm muscle. For simplicity, we do not show any blood volume changes in the simulation (Q_a _= Q_v_). The sustaining recovery middle and high blood flow periods require for 26 and 13 seconds after the exercise respectively in order for the reoxygenation to reach beyond the resting values. The half time of reoxygenation in the middle to high blood flow become 20 seconds and 5 seconds respectively. The reoxygenation half time of 20 seconds are similar to the previous data when blood flow was 4 times of the resting [23]. There is a delayed onset of the reoxygenation; 10, 5 and a few seconds of delay, observed in the exercise simulation, under 1, 4 and 10 times of resting blood flow cases (Figure 5A, 5B, 5C respectively). The delayed onset of reoxygenation is not seen in the ischemic cases.

## Discussion

### The advantages and disadvantages of the method of blood flow measurement

We introduce a novel method to continuously measure arterial blood flow (Q_a_) and venous flow (Q_v_) through the NIRS signal of Hb and HbO_2_. We then demonstrate feasibility of use of the new method during muscle ischemia and exercise performance. This method does not require additional devices or maneuvers since information provided by NIRS is sufficient to calculate blood flow. Therefore, this is a novel, non-invasive and continuous method of blood flow that can be used in many settings of muscle sports exercise as well as in clinical application in real time. This method adds to the importance of NIRS since it provides oxygen concentration information as well as blood flow and oxidative metabolic rate in the muscles. This information will provide major physiological parameters and contribute to better understanding of the skeletal muscle metabolism and circulation status in healthy and diseased muscles.

**Figure 6:**
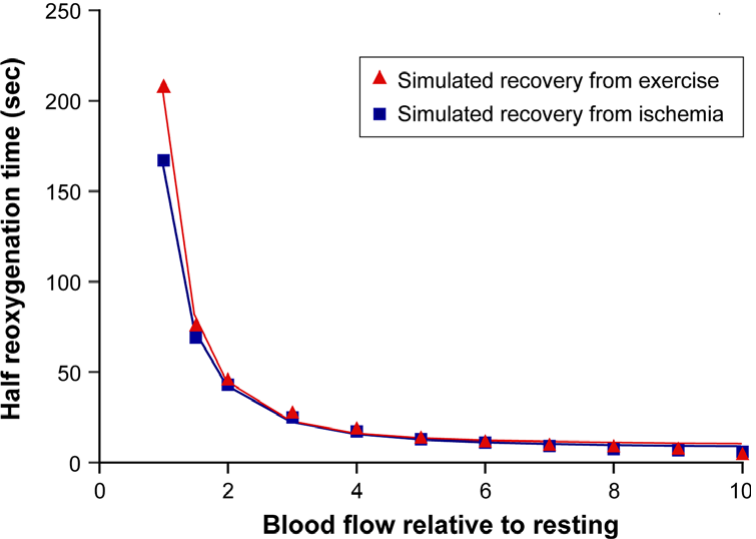
Simulated relationship between blood flow during the recovery and half recovery time of oxygenation in the 3 minutes cuff ischemia (blue line with rectangle) and in the 25%MVC handgrip exercise (red line with triangle) in the finger flexor muscle. Fitted line shows half reoxygenation (sec) = 158.9/(Blood flow)^-2.19 ^+ 7.2 for recovery from cuff ischemia, and half reoxygenaiton (sec) = 197.7/(Blood flow)^-2.49 ^+ 9.2, for post exercise period.

In addition to the advantages of this method discussed above, we can use the described equations as a simulation tool to explain cases of high or low deoxygenation during perturbations and/or recovery of reoxygenation. This is very important in the diagnosis of vascular disease and myopathy [3,27,31-33], as well as when studying different types of muscle fibers and metabolic processes involved [30]. Examples of simulation that can be used to interpret the reoxygenation kinetic in the ischemia studies, as well as recovery from muscle exercise protocols, are demonstrated. In both ischemia and a light exercise models, it is clear that recovery from deoxygenated muscle needs greater blood flow than resting flow, regardless of prior muscle conditions, because resting blood flow is only sufficient to maintain steady state of the same level of oxygenation. Then, we investigate the relationship between blood flow and half-time reoxygenation in recovery from ischemia and the light exercise. Various blood flows in recovery are simulated and summarized in Figure 6. The results show that both the ischemia and the light exercise models have reciprocal relationships between blood flow and reoxygenation, showing that reoxygenation is generally a good indicator of increased blood flow in those models. Note that the differences between the two models are primarily caused by the small oxygen debt treated in the light exercise case, where we input the O_2 _debt to be 10% of the total O_2 _expenditure during the light exercise occurred for 20 seconds. In the exercise model, there is a delayed onset of reoxygenation and the slower reoxygenation half time in the recovery than that of ischemia model (Figure 6). This phenomenon has been seen in many previous studies [30], and greater oxygen debt relative to their blood flow might be the case to explain the slower reoxygenation. This issue should be more carefully investigated in future, but is beyond the scope of this feasibility investigation.

Another feature of the new method is that both arterial and venous blood flow (Q_a _and Q_v_), can be obtained separately. The NIRS is very sensitive to the differences of Q_a _and Q_v _by the blood volume differential information, therefore, it is necessary to separate these inflow and outflow measurements in order to obtain accurate blood flow measurements. To our knowledge, this method is the first to give both Q_a _and Q_v _separately from muscle regional blood flow measurements. Therefore, there has been no previous publication to compare both Q_a _and Q_v _with our data. However, the results from our data generally agree with previous studies [23,25-28].

A two-compartment model of hemoglobin distribution is necessary to get measurements of blood flow, Q_a _and Q_v_. However, hemoglobin distributes arteries, capillaries and venous vessels, and oxygen concentration changes constantly from small arterioles to the end of NIRS visible veins. The two-compartment model of hemoglobin location determines only two average values of [HbO_2_] and [Hb]. The arterial compartment is sequestered in the equation, and any involvement of changes in saturation or blood volume is interpreted as occurring in the venous compartment. If the biological system can not hold the two-compartment model, then the blood flow measurement may result in some error. For example, the arterial compartment may increase its capacity and will then reflect arterializations of the venous compartment. However, one may consider that the arteriole can transport oxygen and can, therefore, be treated as venous in our two-compartment model. But, our definition of the "venous compartment" is a compartment, where the existing blood volume changes its contents by diffusion or convection, therefore this includes arteriole that are capable of exchanging oxygen. Further, we treat this venous volume in the venous compartment as one mixed blood volume with an average value of Hb, and HbO_2_, even though we know there must be decreasing saturation along the capillary length from arterial side to venous side [34,35]. In spite of the oversimplification of the biological system, the agreements of our results to others may come from the fact that the NIRS is much more sensitive to smaller vessels than to larger ones [11]. The difference between arterial and venous compartment here is not anatomical, but the functionality of the vessels, where the artery is only carrying saturated blood to the venous compartment, while the venous system can transport oxygen by diffusion and convection.

### The specific nature of the continuous blood flow measurement using NIRS

NIRS measures the microvasculature rather than the anatomical distribution of the vessels [11] which makes this blood flow measurement more useful in regional muscles. Therefore, we expect the outcomes of the blood flow calculations may differ from conventional techniques. Hemoglobin is used as a probe to measure the blood flow, therefore any discrepancy concerning red cell flow versus plasma flow and red cell flow in the capillary versus large vessel flow will result in a difference in blood flow values. Van Beekvelt noted that the NIRS gave a lower blood flow measurement than the plethysmograph. Using NIRS information to measure lower blood flow outcomes as opposed to measuring larger vessels creates a few concerns. First, the red cell flows five times slower in the capillary [36] than in larger vessels. Second, the hematocrit is 20% less than that in the blood of larger vessels [37]. Third, the red cells cross capillaries intermittently while plasma flow is continuous [38]. Finally, since NIRS visible vessels are smaller ones, its low hematocrit and lower flow rate result in a blood flow calculation smaller than total blood flow [39].

For the purposes of the blood flow measurement, this paper does not differentiate between myoglobin (Mb) and Hb. However, myoglobin is present in muscles. The molar contribution of the Mb in the NIRS is estimated at not more than 50% and the optical equivalency is 20% [40] which comprises a large contribution in the NIRS. Our total hemoglobin concentration in the arm muscles is 98.1 μM/kg. Assuming 12.8 g/100 ml of whole blood (12.8*10/64 mM) and comparing to the whole blood hemoglobin concentration, we can calculate blood concentration in the arm muscles to be approximately 5%. This is reasonably close to the tissue blood concentration; therefore we do not expect a high Mb contribution. With the fact that total arm hemoglobin is underestimated by the partial volume problem, the underneath tissue of the NIRS probe, namely fat and skin will contribute to the lower estimated blood concentration, there **is **as much as 20% of Mb in the muscles. Mb is ignored in the above equations by considering oxygen capacity in the blood and myocytes as one compartment. In addition, oxygen saturation in the Mb is treated as Hb in the capillary. Therefore, the blood flow may have been overestimated as the Mb contribution was taken into account when the muscle becomes very hypoxic and Mb becomes desaturated. In light exercise including less than 3 minutes cuff ischemia, we did not have significant desaturation of Mb [39]. Since Hb and Mb can parallel each other [41,42] and the oxygen gradient, the timing of the changes in blood flow may cause overestimation of the blood flow in response to low tissue saturation when the Mb desaturation occurs.

**Figure 7:**
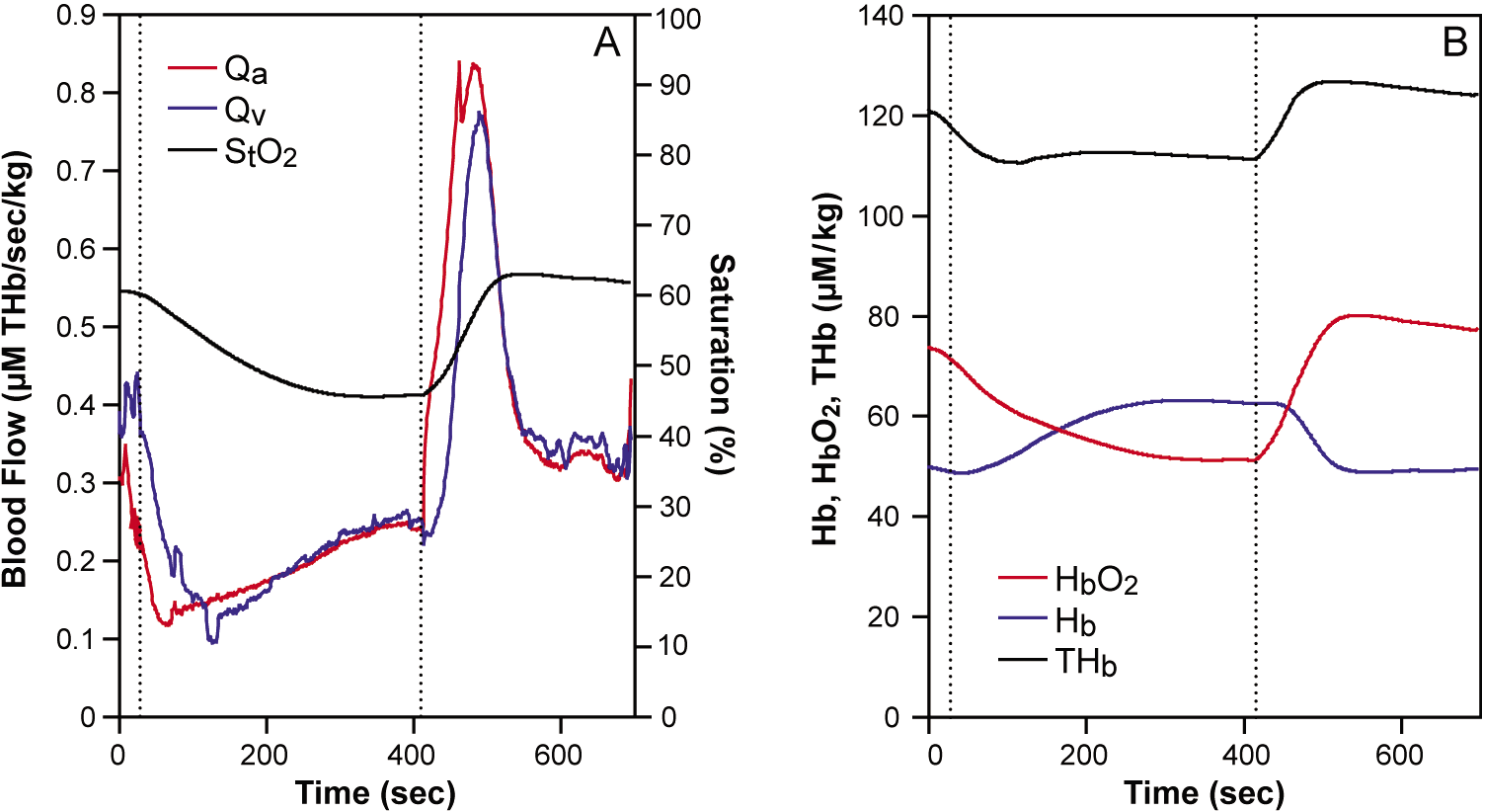
Experimental data of incomplete arm cuff ischemia (between lines). (A), Q_a_: red, Q_v_: blue (THb μM/sec/kg) %S_t_O_2_: (black), (B), HbO_2_: red, Hb: blue, BV: black (μM/kg).

Taking into account of these differences, this measurement method can be used to describe blood flow in a low flow situation. Figure 7 describes a case where a very small blood flow can be detected that may be hard to measure accurately by other modalities. In Figure 7, incomplete cuff ischemia was imposed in an arm at 160 mmHg, and gradual increases in Q_a _and Q_v _are observed under the cuff, and hemoglobin saturation becomes steady state at 5 min of cuff ischemia. Even though the blood flow is very slow, we can observe increasing blood flow, showing the physiological adaptation of the arm circulation and the sensitivity and strength of this technique of measuring the blood flow.

Figure 7 also demonstrates that the Q_a _and Q_v _are not always simultaneous – actual drainage of venous blood occurred at the beginning of the cuff, and at the end, arterial blood into the muscle and venous outflow is delayed until the muscle is adequately filled with the blood. This indicates the possibility that detected inflow and outflow can be used to better understand physiological functions during exercise tests.

The most common local muscle blood flow measurement method is the ultrasound Doppler blood flow [13]. It is used on larger muscle groups since it obtains information from larger diameter vessels such as the brachial artery [25] and the femoral artery and produces volume per second quantity from the velocity. The method provides information on oxygen transport of entire muscle groups or complete exercise effects [43-45]. On the other hand, the NIRS techniques provide more regional muscle blood flow using a venous occlusion technique [7], and Hb, HbO_2 _from NIRS data. However the venous or arterial occlusion techniques are not a continuous measurement and can disturb the exercise performance. This new method presented in this paper focuses on regional blood flow and provides a continuous measurement as well as in real time. Moreover, it overcomes challenging situations when additional equipment and manipulation are not possible. This technique adds to the importance of NIRS since data may be interpreted by examining the relationship between tissue oxygenation changes and blood flow. This is important for diagnostic purposes and the interpretation of muscle performance and physiology.

The equations discussed above prove to be useful in the calculation of blood inflow and outflow. Since O_2 _must be known in order to analyze blood flow data, accuracy of the blood flow measurement is dependent upon O_2 _information. If O_2 _is not measured, then assumptions can be made from previous literature, as we did in the 25% handgrip exercise case (Figure 2). If the assumption of O_2 _is close enough, then the blood flow values are correct. Since we do not know how different these O_2 _are between subjects, the accuracy can not be examined in this paper. When we seek higher accuracy, we must design experiments where we can measure O_2 _either simultaneously or at another time in the same subject. Simultaneous O_2 _measurement using an ischemia protocol is shown in Figure 1 and 3. Another assumption required, is S_v_O_2_. This is important to provide [HbO_2_]_a _and [HbO_2_]_v_. We used arterial venous blood ratio of 28.3%/71.7%, calculated from S_t_O_2 _(our measurement) and S_v_O_2 _[46] to calculate [HbO_2_]_v _and [HbO_2_]_a _in this paper. However, this assumption is only reliable in healthy subjects since we do not know if diseased persons or elite athletes have different values. S_v_O_2 _measurement from venous blood sampling may be a good solution, however the venous blood sampling be contaminated from other tissues, such as anastomosis and skin and bone. The majority of venous blood from the muscles consists of a mixture of blood from the capillary and shunting vessels, therefore its' saturation is higher than that of capillary outflow. In this case, it may be more reliable to use S_v_O_2 _from non-invasive venous occlusion technique [46,47] in order to measure more accurate mirovascular targeted blood flow.

Another issue concerns resting [Hb], [HbO_2_] which are required in the calculation. The absolute amount of [Hb], [HbO_2_] can be measured by TRS, Frequency Domain spectroscopy, as well as CWS with multi detection or multi wavelength methods. However resting [Hb] and [HbO_2_] in the human muscles have been measured by only a few researchers ([48], this paper). It is possible to use assumed values (Table 1) in the healthy individuals. However we will have to validate blood flow values.

## Summary and conclusion

We introduce a novel blood flow measurement method for non-invasive and continuous regional blood flow monitoring through the NIRS signal. This method uses differential information of muscle Hb and HbO_2 _and can be monitored in real time. We demonstrate feasibility of the method by showing measured blood flow time course during ischemia and light exercise on arm muscle, and by simulating these two models using the equations upon which the method is based. These blood flow time profiles agree with previous data as well as each other. Thus we conclude that this method is available to use for muscle activity studies in future.

## References

[B1] Hampson NB, Jobsis-VanderVliet FF, Piantadosi CA (1987). Skeletal muscle oxygen availability during respiratory acid-base disturbances in cats. Respir Physiol.

[B2] De Blasi RA, Cope M, Elwell C, Safoue F, Ferrari M (1993). Noninvasive measurement of human forearm oxygen consumption by near infrared spectroscopy. Eur J Appl Physiol Occup Physiol.

[B3] McCully KK, Halber C, Posner JD (1994). Exercise-induced changes in oxygen saturation in the calf muscles of elderly subjects with peripheral vascular disease. J Gerontol.

[B4] Chance B, Dait M, Zhang C, Hamaoka T, Hagerman F (1992). Recovery from exercise-induced desaturation in the quadriceps muscle of elite competitive rowers. Am J Physiol Cell Physiol.

[B5] Szmedra L, Im J, Nioka S, Chance B, Rundell KW (2001). Hemoglobin/myoglobin oxygen desaturation during Alpine skiing. Med Sci Sports Exerc.

[B6] Rundell KW, Nioka S, Chance B (1997). Hemoglobin/myoglobin desaturation during speed skating. Med Sci Sports Exerc.

[B7] Homma S, Eda H, Ogasawara S, Kagaya A (1996). Near-infrared estimation of O_2 _supply and consumption in forearm muscles working at varying intensity. J Appl Physiol.

[B8] Hamaoka T, Iwane H, Shimomitsu T, Katsumura T, Murase N, Nishio S, Osada T, Kurosawa Y, Chance B (1996). Noninvasive Measures of oxidative metabolism on working human muscles by near-infrared spectroscopy. J Appl Physiol.

[B9] Sako T, Hamaoka T, Higuchi H, Kurosawa Y, Katsumura T (2001). Validity of NIR spectroscopy for quantitatively measuring muscle oxidative metabolic rate in exercise. J Appl Physiol.

[B10] DeLorey DS, Kowalchuk JM, Paterson DH (2005). Adaptation of pulmonary O2 uptake kinetics and muscle deoxygenation at the onset of heavy-intensity exercise in young and older adults. J Appl Physiol.

[B11] Liu H, Chance B, Hielscher AH, Jacques SL, Tittel FK (1995). Influence of blood vessels on the measurement of hemoglobin oxygenation as determined by time-resolved reflectance spectroscopy. Med Phys.

[B12] Van BeekBelt M, Colier WNJM, Wevers RA, van Engelen BGM (2001). Performance of near-infrared spectroscopy in measuring local O_2 _consumption and blood flow in skeletal muscle. J Appl Physiol.

[B13] Wesche J (1986). The time course and magnitude of blood flow changes in the human quadriceps muscles following isometric contraction. J Physiol.

[B14] Frank LR, Wong EC, Haseler LJ, Buxton RB (1999). Dynamic imaging of perfusion in human skeletal muscle during exercise with arterial spin labeling. Magn Reson Med.

[B15] Raitakari M, Nuutila P, Ruotsalainen U, Laine H, Teras M, Iida H, Makimattila S, Utriainen T, Oikonen V, Sipila H, Haaparanta M, Solin O, Wegelius U, Knuuti J, Yki-Jarvinen H (1996). Evidence for dissociation of insulin stimulation of blood flow and glucose uptake in human skeletal muscle: studies using [^15 ^O]H_2 _O, [18F]fluoro-2-deoxy-D-glucose, and positron emission tomography. Diabetes.

[B16] Nioka S, Chance B, Nakayama K (1998). Possibility of monitoring mitochondrial activity in isometric exercise using NIRS. Adv Exp Med Biol.

[B17] Nioka S, Wen S, Zhang J, Du J, Intes X, Zhao Z, Chance B (2006). Simulation study of breast tissue hemodynamics during pressure perturbation. Adv Exp Med Biol.

[B18] Yang Y, Liu H, Li X, Chance B (1997). Low-cost frequency-domain photon migration instrument for tissue spectroscopy, oximetry and imaging. Opt Eng.

[B19] Yu G, Durduran T, Lech G, Zhou C, Chance B, Yodh AG (2005). Time-dependent blood flow and oxygenation in human skeletal muscles measured by noninvasive near-infrared diffuse optical spectroscopies. Journal of Biomedical Optics.

[B20] Agewall S, Whalley GA, Doughty RN, Sharpe N (1999). Handgrip exercise increases post occlusion hyperemic brachial artery dilatation. Heart.

[B21] Sevick EM, Chance B, Leigh J, Nioka S, Maris M (1991). Quantitation of time- and frequency-resolved optical spectra for the determination of tissue oxygenation. Anal Biochem.

[B22] Lin Y, Lech G, Nioka S, Intes X, Chance B (2002). Noninvasive, low-noise, fast imaging of blood volume and deoxygenation changes in muscles using light-emitting diode continuous-wave imager. Rev Sci Instrum.

[B23] Van Beekvelt MCP, Shoemaker JK, tschakovsky ME, Hopman MTE, Hughson RL (2001). Blood flow and muscle oxygen uptake at the onset and end of moderate and heavy dynamic forearm exercise. Am J Physiol.

[B24] Costes F, Barthelemy JC, Feason L, Busso T, Geyssant A, Denis C (1995). Comparison of muscle near infrared spectroscopy and femoral blood gases during steady-state exercise in humans. J Appl Physiol.

[B25] Osada T, Katsumura T, Murase N, Sako T, Higuchi H, Kime R, Hamaoka T, Shimomitsu T (2003). Post-exercise Hyperemia after Ischemic and Non-ischemic Isometric Handgrip Exercise. J Physiol Anthropol Appl Human Sci.

[B26] Banitt PF, Smits P, Williams SB, Ganz P, Creager MA (1996). Activation of ATP-sensitive potassium channels contributes to reactive hyperemia in humans. Am J Physiol.

[B27] McCully KK, Smith S, Rajaei S, Leigh JS, Natelson BH (2003). Blood flow and muscle metabolism in chronic fatigue syndrome. Clin Sci.

[B28] Bangsbo J, hellsten Y (1998). Muscle blood flow and oxygen uptake in recovery from exercise. Acta Physiol Scand.

[B29] Kagaya A, Homma S (1997). Brachial arterial blood flow during static handgrip exercise of short duration at varying intensities studied by a Doppler ultrasound method. Acta Physiol Scand.

[B30] Kime R, Hamaoka T, Sako T, Murakami M, Homma T, Katsumura T, Chance B (2003). Delayed reoxygenation after maximal isometric handgrip exercise in high oxidative capacity muscle. Eur J Appl Physiol.

[B31] Salman M, Glantzounis GK, Yang W, Myint F, Hamilton G, Seifalian AM (2005). Measurement of critical lower limb tissue hypoxia by coupling chemical and optical techniques. Clin Sci.

[B32] Hayden RE, Tavill MA, Nioka S, Kitai T, Chance B (1996). Oxygenation and blood volume changes in flaps according to near-infrared spectrophotometry. Arch Otolaryngol Head Neck Surg.

[B33] McCully KK, Smith S, Rajaei S, Leigh JS, Natelson BH (2004). Muscle metabolism with blood flow restriction in chronic fatigue syndrome. J Appl Physiol.

[B34] Piiper J, Sheid P (1986). Cross-sectional PO2 distributions in Krogh cylinder and solid cylinder models. Respir Physiol.

[B35] Honig CR, Gayeski TE, Clark A, Clark PA (1991). Arteriovenous oxygen diffusion shunt is negligible in resting and working gracilis muscles. Am J Physiol.

[B36] Zweifach BW (1974). Quantitative studies of microcirculatory structure and function, I: analysis of pressure distribution in the terminal vascular bed in cat mesentery. Circ Res.

[B37] Pries AR, Ley K, Gaehtgens P (1986). Generalization of the Fahraeus principle for microvessel networks. Am J Physiol.

[B38] Tomita M, Schiszler I, Takeda H, Tomita Y, Osada T, Unekawa M, Tanahashi N, Suzuki N (2005). Spatial and temporal heterogeneity of single capillary plasma flow and RBC tracking in the rat cerebral cortex. Journal of Cerebral Blood Flow & Metabolism.

[B39] Appelgren KL (1972). Effect of perfusion pressure and hematocrit on capillary flow and transport in hyperemic skeletal muscle of the dog. Microvasc Res.

[B40] Nioka S, Wang DJ, Im J, Hamaoka T, Wang ZJ, Leigh JS, Chance B (2006). Simulation of Mb/Hb in NIRS and oxygen gradient in the human and canine skeletal muscles using H-NMR and NIRS. Exp Med Biol.

[B41] Gayeski TE, Honig CR (1991). Intracellular PO_2 _in individual cardiac myocytes in dogs, cats, rabbits, ferrets, and rats. Am J Physiol.

[B42] Chance EM (1988). Estimation of the magnitude of oxygen gradients in cardiac muscle in the coherent state. J Appl Cardiology.

[B43] Wolf U, Wolf M, Choi JH, Levi M, Choudhury D, Hull S, Coussirat D, Paunescu LA, Safonova LP, Michalos A, Mantulin WW, Gratton E (2003). Localized irregularities in hemoglobin flow and oxygenation in calf muscle in patients with peripheral vascular disease detected with near-infrared spectrophotometry. J Vasc Surg.

[B44] Laaksonen MS, Kalliokoski KK, Kyröläinen H, Kemppainen J, Teräs M, Sipilä H, Nuutila P, Knuuti J (2003). Skeletal muscle blood flow and flow heterogeneity during dynamic and isometric exercise in humans. Am J Physiol.

[B45] Mizuno M, Kimura Y, Iwakawa T, Oda K, Ishii K, Ishiwata K, Nakamura Y, Muraoka I (2003). Regional differences in blood flow and oxygen consumption in resting muscle and their relationship during recovery from exhaustive exercise. J Appl Physiol.

[B46] Yoxall CW, Weindling AM (1997). Measuremnets of venous oxyhemoglobin saturation in the adult human forarm by near infrared spectroscopy wityh venous occlusion. Med Biol Eng Comput.

[B47] Franceschini MA, Boas DA, Zourabian A, Diamond SG, Nadgir S, Lin DW, Moore JB, Fantini S (2002). Near-infrared spiroximetry: noninvasive measurements of venous saturation in piglets and human subjects. J Appl Physiol.

[B48] Hamaoka T, Katsumura T, Murase N, Nishio S, Osada T, Sako T, Higuchi H, Kurosawa Y, Shimomitsu T, Miwa M, Chance B (2000). Quantification of ischemic muscle deoxygenation by near infrared time-resolved spectroscopy. J Biomed Opt.

